# Exploration of the application of augmented reality technology for teaching spinal tumor’s anatomy and surgical techniques

**DOI:** 10.3389/fmed.2024.1403423

**Published:** 2024-07-10

**Authors:** Shuzhong Liu, Jianxin Yang, Hui Jin, Annan Liang, Qi Zhang, Jinyi Xing, Yong Liu, Shuangshou Li

**Affiliations:** ^1^Department of Orthopedic Surgery, Peking Union Medical College Hospital, Peking Union Medical College and Chinese Academy of Medical Sciences, Beijing, China; ^2^Fundamental Industry Training Center, Tsinghua University, Beijing, China

**Keywords:** augmented reality technology, spinal tumors, anatomy teaching, surgical teaching, teaching evaluation

## Abstract

**Background:**

Augmented reality (AR) technology is gradually being applied in surgical teaching as an innovative teaching method. Developing innovative teaching methods to replicate clinical theory and practical teaching scenarios, simulate preoperative planning and training for bone tumor surgery, and offer enhanced training opportunities for young physicians to acquire and apply clinical knowledge is a crucial concern that impacts the advancement of the discipline and the educational standards for young orthopedic physicians.

**Objective:**

This study explores the application effect of augmented reality technology in anatomy teaching and surgical clinical teaching for spinal tumor.

**Methods:**

The method utilizes virtual reality and augmented reality technology to present a spinal tumor model and the surgical process of percutaneous vertebroplasty. We conducted a random selection of 12 students forming into the augmented reality teaching group and 13 students forming into the traditional teaching group among the 8-year medical students from Peking Union Medical College and Tsinghua University, ensuring that the age and learning stage of the students in both groups were similar. Two groups of students were taught using traditional teaching methods and augmented reality technology-assisted teaching methods, respectively. A questionnaire survey was conducted after class to assess the quality of course instruction, student motivation in learning, their proficiency in anatomical structures, their comprehension of spinal tumor growth and metastasis, and their understanding and proficiency in percutaneous vertebroplasty.

**Results:**

This study was the first to apply augmented reality technology in teaching, using spinal tumors and percutaneous vertebroplasty as examples, a head-mounted augmented reality device was used to create learning scenarios, presenting the complex three-dimensional spatial structure intuitively. The two groups of students differ significantly in their rating of teaching quality, enthusiasm for learning, knowledge of anatomical features, understanding of spinal trabecular structure, and understanding of steps in percutaneous vertebroplasty. The augmented reality technology-assisted teaching system demonstrates outstanding advantages.

**Conclusion:**

Augmented reality technology has great potential and broad prospects in teaching bone tumors, which can help improve the visualization, interactivity, and three-dimensional spatial sense of medical teaching in spinal tumor. The application and development prospects of using augmented reality technology for anatomy instruction, surgical teaching, and simulation training are extensive.

## Background

In the past decade, extended reality (XR) technology has developed exponentially, overlaying virtual reality (VR) onto the real-world environment to display information and data at various levels of the reality-virtuality continuum (1, 2). Augmented reality (AR) technology combines computer-generated three-dimensional (3D) virtual models with real-world scenes to achieve real-time interaction among users, the physical environment, and digital content. It has gradually been implemented in medical clinical teaching and practice (3, 4). Multiple studies have demonstrated that augmented reality technology, as a cutting-edge instructional approach, can be applied in orthopedic teaching, encompassing sub-specialties like trauma, joint surgery, spinal surgery, and sports medicine (5–10). This application can potentially enhance the caliber and efficacy of course instruction.

The knowledge and theory related to bone and soft tissue tumor surgery are complex, the skill learning curve is long, and students have limited practical opportunities. Conventional instructional approaches pose challenges in resolving numerous real-world issues, while AR technology integrates virtual and real environments. Providing students with an immersive learning experience in bone tumor surgery can yield several benefits, including improved teaching efficiency, increased student engagement, and enhanced course satisfaction (11, 12). Furthermore, some studies have confirmed that AR technology can simulate clinical theory and practical teaching scenarios, simulate preoperative planning and training for bone tumor surgery, and provide valuable opportunities for young physicians to learn and practice clinical knowledge, which is conducive to promoting the reform and development of traditional bone tumor surgery teaching (13, 14). This study aims to elucidate the practical significance of utilizing augmented reality technology in instructing spinal tumor anatomy and surgical clinical teaching for the first time. Simultaneously, the advantages and disadvantages of this technology application in teaching practice are summarized, and the future development trend of AR technology is thoroughly discussed.

## Materials and methods

### Basic materials

**Figure 1:**
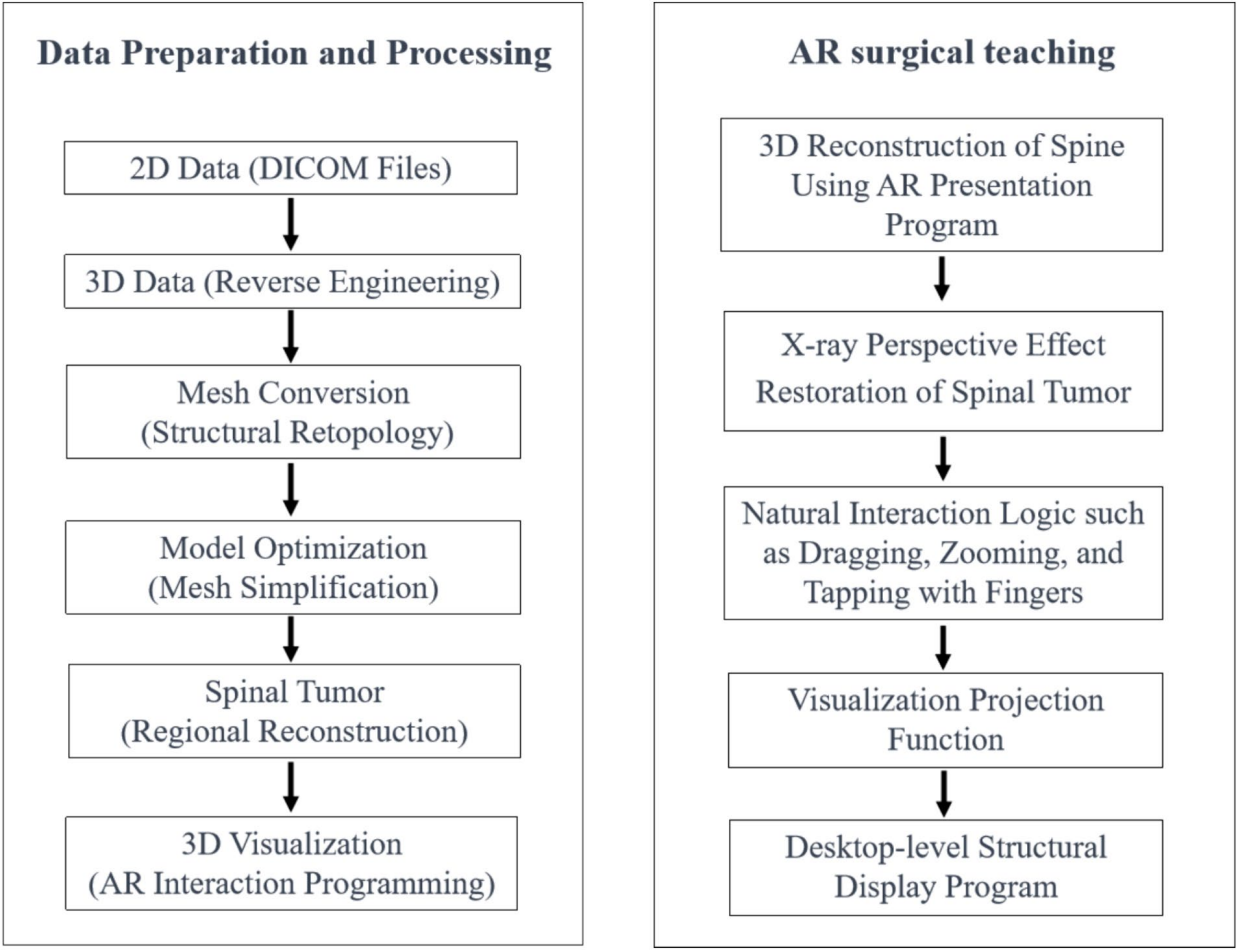
Schematic diagram of the application of augmented reality technology in this study.

**Figure 2:**
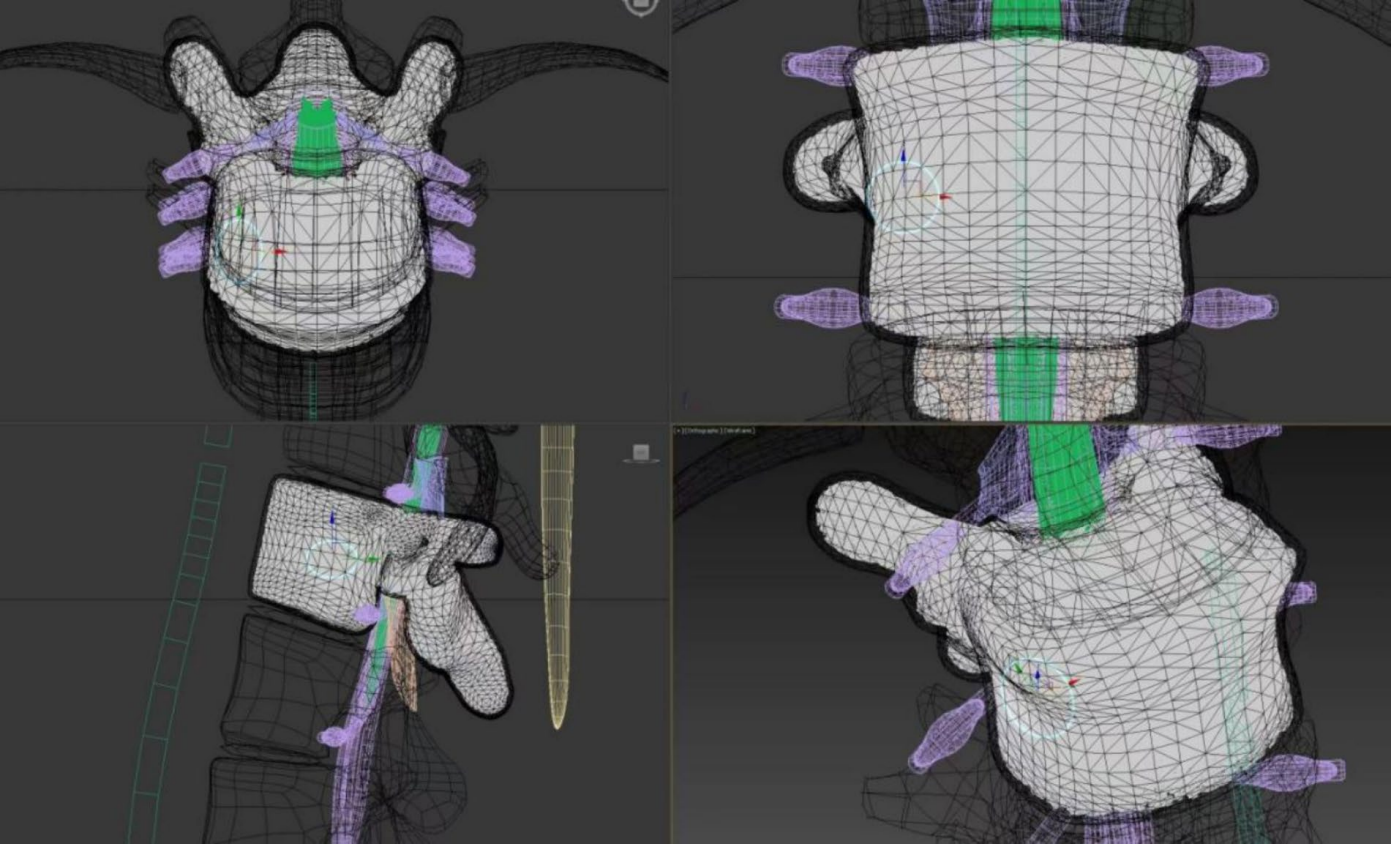
Schematic diagram of early reconstruction of spinal tumor model.

**Figure 3:**
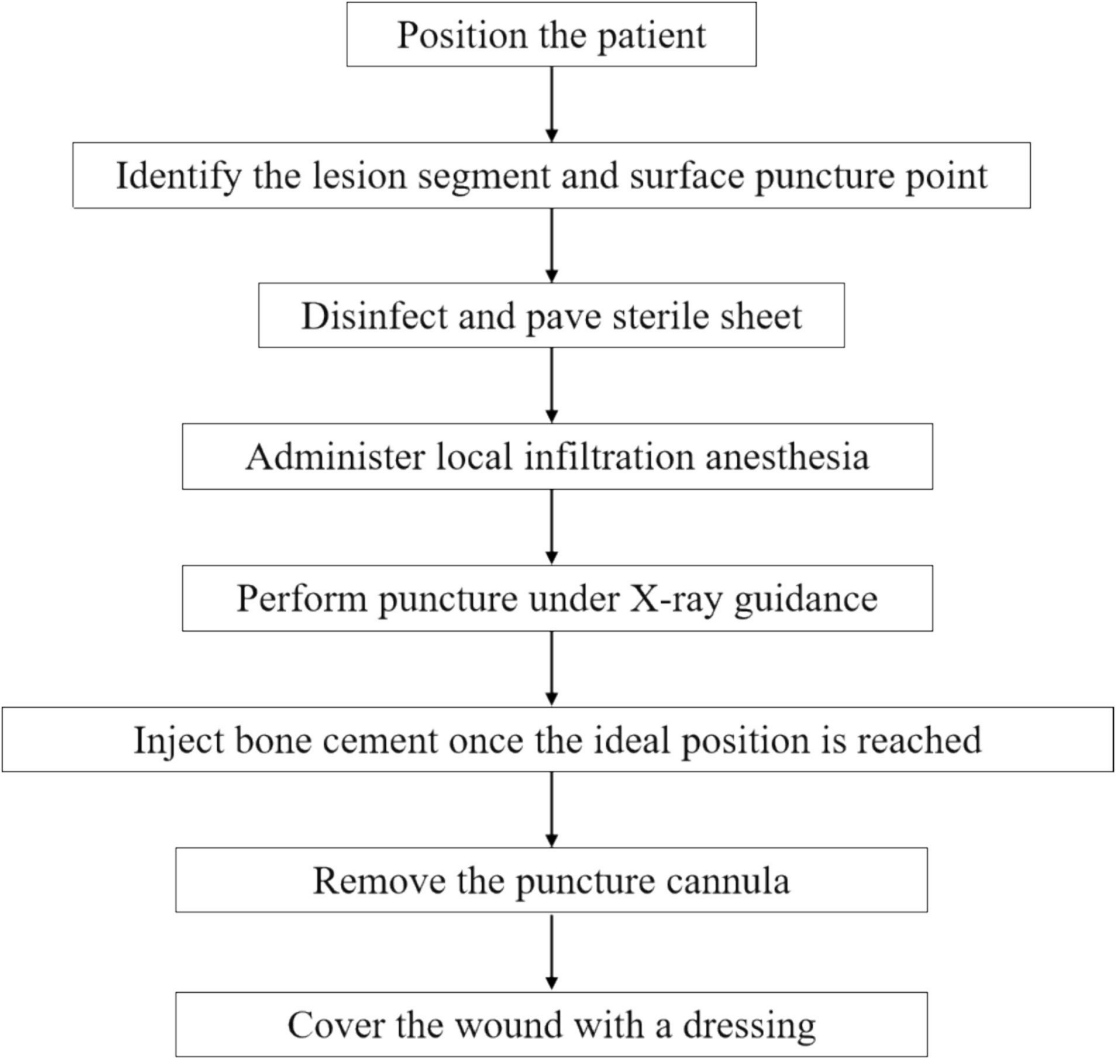
The simplified flowchart of the PVP procedure steps.

During the initial preparation process of this study, the orthopedic bone and soft tissue tumor team at Peking Union Medical College Hospital provided relevant data. The medical team exemplified surgical instruments used for spinal tumors and percutaneous vertebroplasty, elucidated the surgical steps, and demonstrated the operative process. Based on this premise, the team from the Fundamental Industry Training Center of Tsinghua University utilized simulation technique to create interactive anatomical program of spinal tumor and surgical operation procedure for percutaneous vertebroplasty (PVP). This primarily involved script development for AR-assisted spinal tumor and percutaneous vertebroplasty surgery, calibrating the position of anatomical structures, measuring and reconstructing surgical instruments, and establishing multi-person collaboration and position sharing (Figure 1). Construction of spinal tumor model: Firstly, with informed consent from the patient and approval from the hospital’s ethics committee, Digital Imaging and Communications in Medicine (DICOM) files were obtained. Since the DICOM files were 2D image files, the creation of the three-dimensional mesh was performed using “3D Slicer” software, which can be considered as reverse engineering. Subsequently, the model was refined and reconstructed using “3Ds Max” software; Secondly, we have taken the following steps to optimize the mesh: (1) Retopology: The mesh topology was optimized; (2) Manual simplification: Unnecessary meshes were removed, and structures with poor display quality were optimized; (3) Structural adjustment: Manually drawn models can have deviations, similar to mechanical assembly errors. Using commands like lattice deformation, details were smoothly adjusted to ensure complete integration of the lumbar vertebrae; (4) Boolean operations: Overlapping parts of two models were removed. For example, independently drawn spinal tumor and vertebral body are separate parts and will overlap when combined. Using Boolean algorithms, appropriate spaces were cut from the vertebral body to accommodate the spinal tumor. Subsequently, we used Unity program to create the AR environment and implement the communication functionality through code, then finally deployed it to HoloLens 2 for each student to experience after multiple debugging. We reconstructed surgical instruments for percutaneous vertebroplasty surgery, includeing drapes, anesthesia syringe, needle, puncture cannula, drill, sampler, bone cement, push rod, gauze, etc. The tasks of the teaching groups were to help students master the anatomical structure of the spine, spinal tumor, and learn the PVP surgical procedure. The above models all have natural interaction functions such as finger dragging, zooming, and tapping, for students to simulate and operate. In addition, the program has a visual projection function, which allows for viewing of spinal tumor lesions and surrounding structures under the guidance of teachers, as well as demonstrating the surgical process. The perspective mode is akin to viewing a real human body, where internal organs and bone structures are not visible beneath the skin. When inserting medical instruments into the human model, students rely on experience and medical imaging references. However, the perspective mode is different. It assists students by providing a three-dimensional perspective view, allowing them to clearly and intuitively see through the skin to observe the vertebral structure and spinal tumor. Through close collaboration, researchers presented spinal tumor and percutaneous vertebroplasty by using augmented reality (Figures 2, 3).

### Student grouping

To further confirm the application effect and feasibility of AR technology in teaching spinal tumor’s anatomy and surgery, 8-year program medical students were selected from Peking Union Medical College and Tsinghua University who met the inclusion criteria in the 8-year clinical medicine program as research subjects. The inclusion criteria for this study are as follows: students who have studied for a minimum of 5 years, and have not previously undergone any spinal tumor surgery or virtual reality/augmented reality-assisted surgery. Exclusion criteria: students who could not complete the 2-h teaching plan on time, could not participate in post-teaching evaluations, were in a grade of fewer than 5 years, had previously participated in spinal tumor surgery, or received surgical simulation training.

### Theoretical teaching

The teacher employs a uniform teaching curriculum to instruct involved students on fundamental theories and anatomical structures of spinal tumor. This encompasses topics such as spinal anatomy, spinal tumor anatomy, prevalent disorders associated with spinal tumors, spinal tumor metastasis, standard surgical procedures, surgical indications and contraindications, details for percutaneous vertebroplasty, and other specialized knowledge. Following the completion of the instruction, a group simulation lesson will be carried out for all students in two groups.

### Group simulation operation teaching

The students included in this study will be randomly divided into a traditional teaching group (Group A, using traditional teaching methods in the simulation lesson) and a AR-assisted teaching group (Group B, using head-worn HoloLens 2 holographic glasses for AR teaching in the simulation lesson). The same senior physician will teach the two groups of students, and the teaching staff will have the same class hours, courseware, basic knowledge explanation, and model teaching aids application, except for AR-related equipment. The teacher in the traditional teaching group (Group A) explained all same procedures using 3D-printed lumbar spine anatomy models, PVP surgical tools, and solid human anatomy models. The students in group B experienced the spinal tumor instance model and PVP surgical operation process using augmented reality technology and head-worn HoloLens holographic glasses. As they go through a spinal tumor instance model, students can change the view from perspective to non-perspective, remove, enlarge, and decrease the vertebrae and attachments of each segment. The layout and structure of the spinal trabeculae, as well as the location and invasive features of spinal tumors, can be intuitively felt by students when they switch to perspective mode. Based on their learning objectives, students can control their learning process, rewatch crucial steps, and switch to non-perspective or perspective modes during the PVP surgical simulation demonstration.

### Investigation and evaluation after teaching

**Figure 4:**
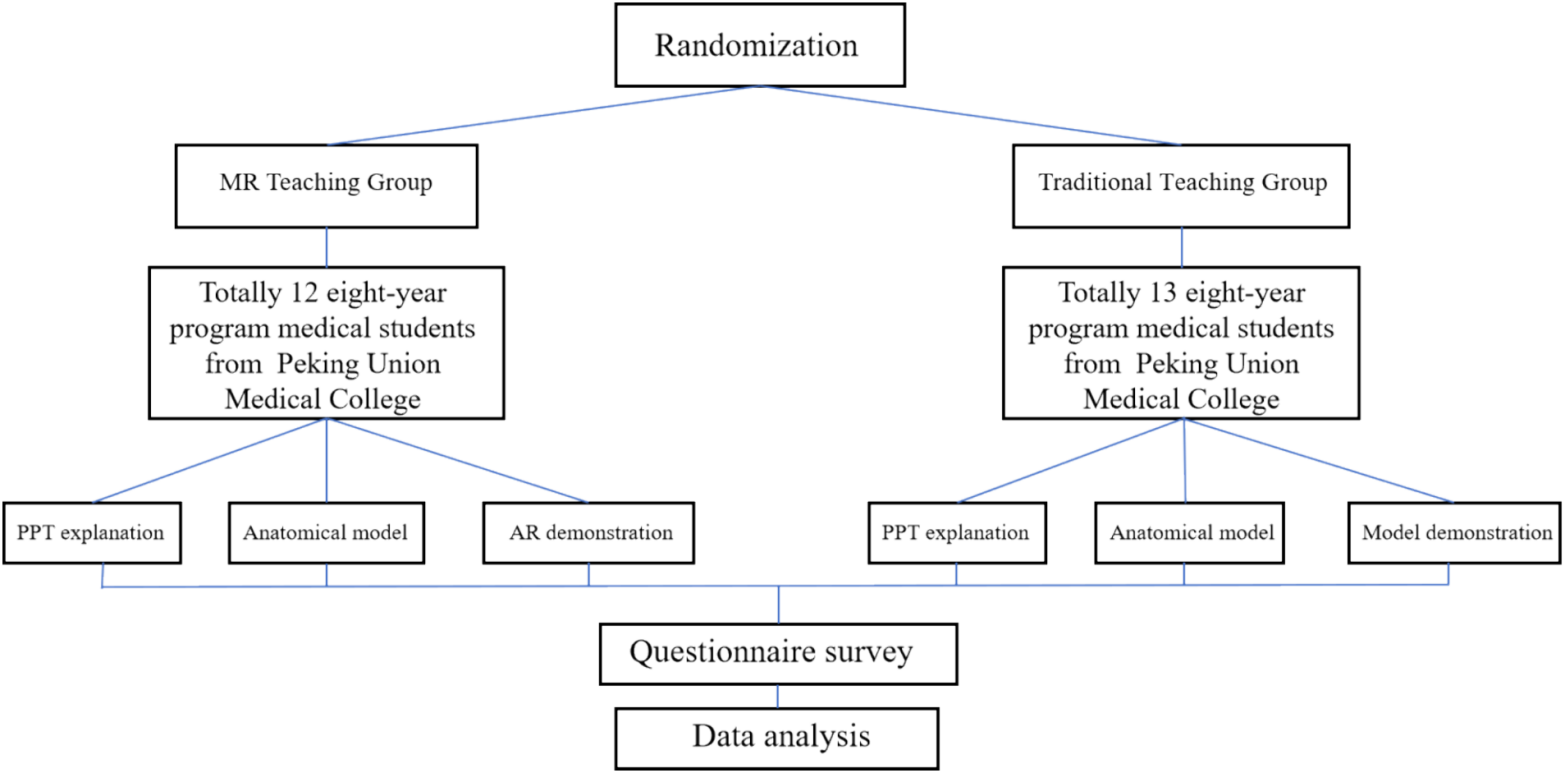
Implementation flowchart of this study.

When the regular academic offerings is over, students complete a survey comparing their levels of initiative, interaction, course outcomes, satisfaction, and information retention to see how the two groups fared. Using a step-by-step score evaluation approach, the questionnaire options are all quantified using scores with a range of 1–10 points or 1–5 points. At the same time, students in both groups were asked to fill out subjective questionnaires outlining their ideas for how the teaching techniques could be improved. After the completion of the regular academic offerings and the completion of the first survey questionnaire, all students in group A received augmented reality teaching (Group C). After completing all learning content, students in group C received the second survey. Two orthopedic experts reviewed the data and findings from the survey and used statistical methods to conclude. The flowchart of this study is shown in Figure 4.

### Statistical analysis

Original data were analyzed using MedCalc 15.2.2 software (MedCalc, Ostend, Belgium). To check if the data follows a normal distribution, the Shapiro–Wilk test was applied. However, considering the particularity of questionnaire surveys, this study used non-parametric tests. The Mann–Whitney independent sample test was used to verify the correlation between the traditional teaching group (Group A) and augmented reality-assisted teaching group (Group B), and the data are described as the median and 25 and 75th interquartile ranges (Q25, Q75). U value, *p* value, and *r* values have been reported. The data are described as the median and 25 and 75th interquartile ranges (Q25, Q75); The effect size (ES) was calculated by dividing the *Z*-score by the square root of *n*: 0.20–0.49 is considered a small effect; 0.50–0.79 is considered a medium effect; and 0.80 and above are considered a large effect (15, 16). The Wilcoxon rank-sum test was used to verify the correlation between the traditional teaching group (Group A) and continuing AR teaching in the traditional group (Group C), and the data are described as the median and 25 and 75th interquartile ranges (Q25, Q75). *Z*-value and *p* values have been reported. *p* < 0.05 was identified as statistically significant. The Wilcoxon rank-sum test were created using SPSS 23.0 (IBM, Armonk, NY, United States). Figures were created using GraphPad Prism 10.0 (GraphPad Software, San Diego, CA, United States).

## Results

### Basic information between groups

The traditional teaching group included 13 students, four males and nine females, who were all eighth-year clinical medical students in fifth grade or sixth grade. The average age of the participants in the traditional teaching group is 23.5 ± 0.9 years old. Twelve medical students (seven males and five females) from fifth grade or sixth grade in the augmented reality technology teaching group served as comparisons. The average age of the students in the augmented reality assisted teaching group is 23.7 ± 1.3 years old. In terms of age, stage of cultivation, etc., no statistically significant difference was found between the two groups. According to the research requirements, all participants fulfilled the inclusion criteria and finished the course, questionnaires, and post-class feedback assessments.

### Differences in teaching effectiveness in different groups

**Figure 5:**
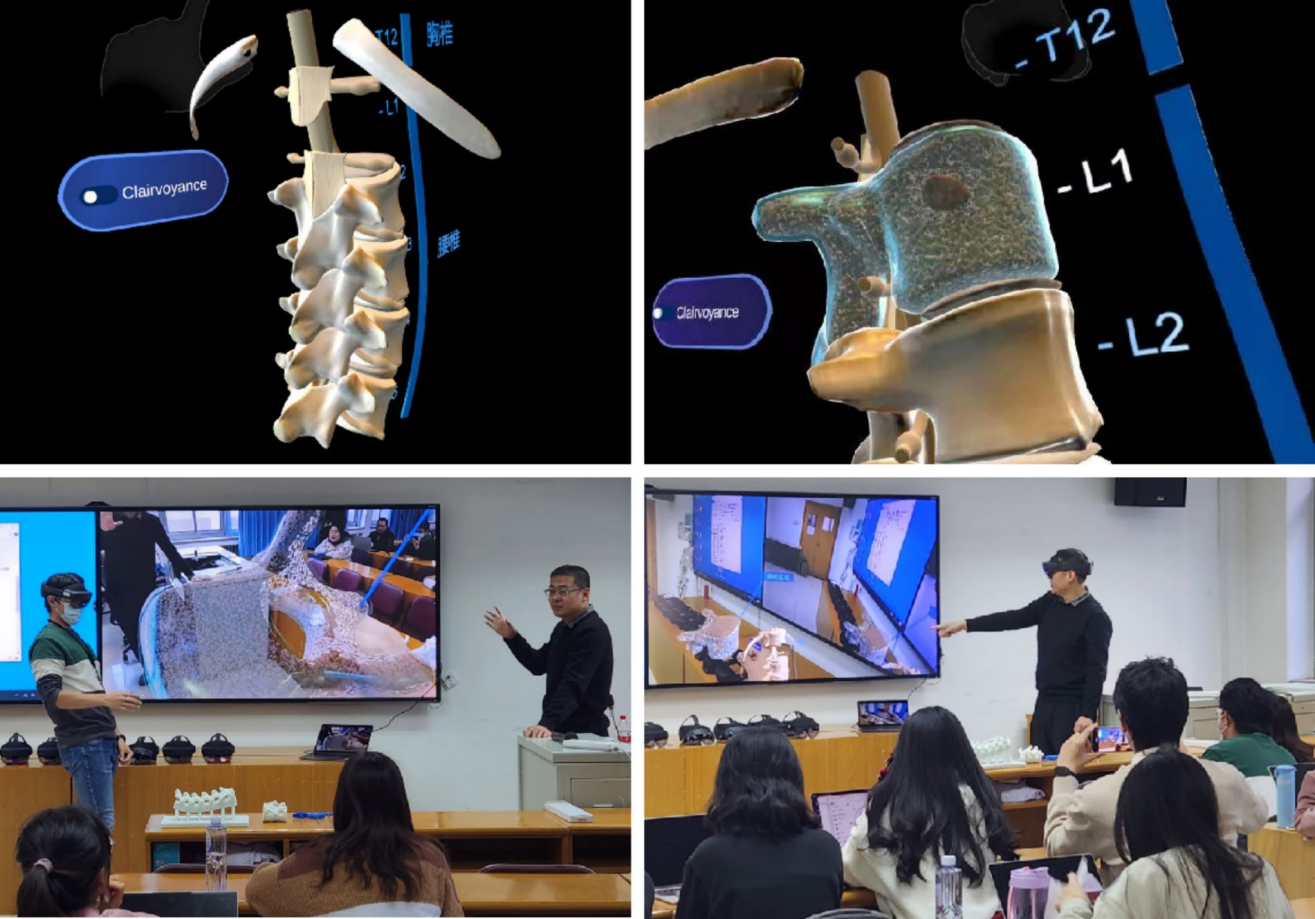
Augmented reality technology displaying the anatomical tumor model in L1 vertebral body.

**Figure 6:**
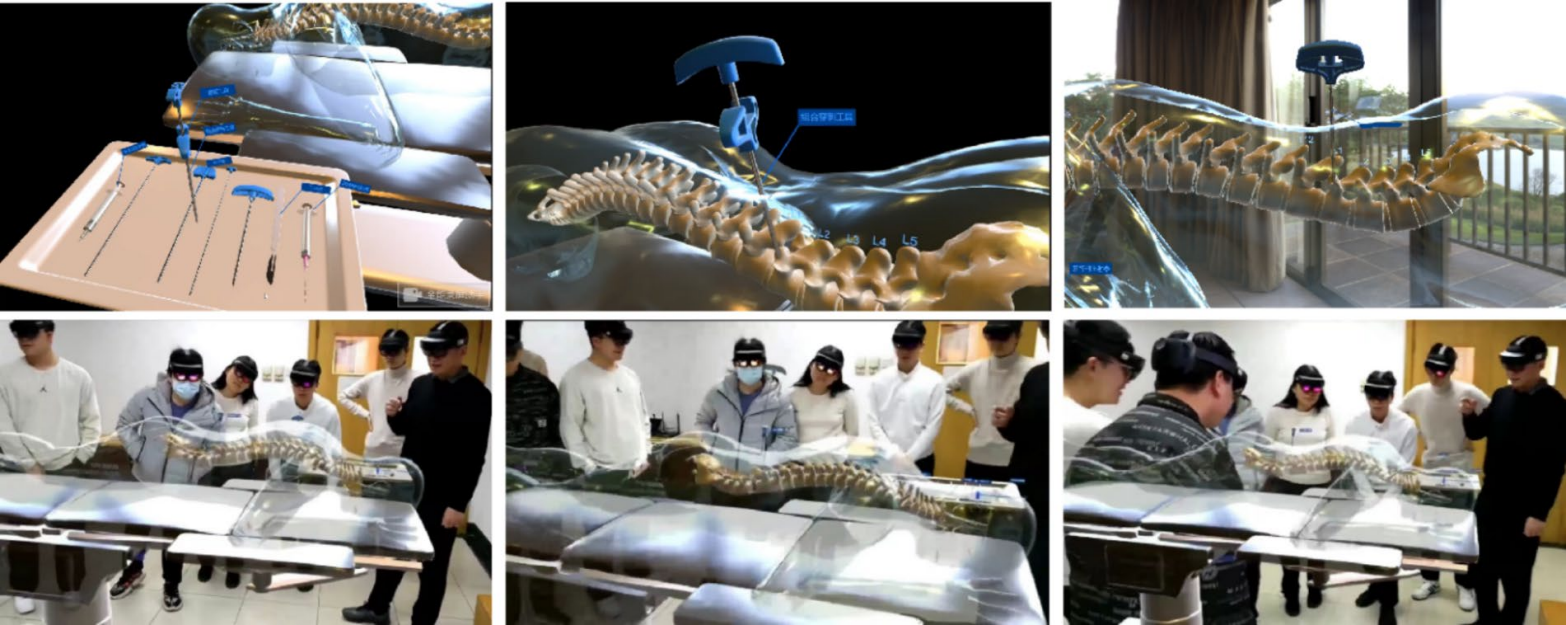
Augmented reality technology displaying the surgical process of percutaneous vertebroplasty (PVP).

**Figure 7:**
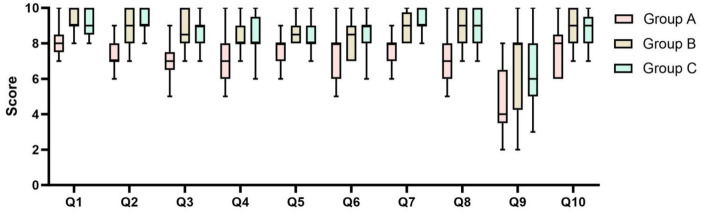
The box plots displaying the distribution differences among three groups. Plot: min to max; Q: Question; Q1: Teaching quality; Q2: Learning interest; Q3: Understanding of spinal anatomy; Q4: Understanding of spinal trabecular structure; Q5: Understanding of the common sites for spinal tumors; Q6: Understanding of the involvement and metastasis of spinal tumors; Q7: Understanding of the general steps of PVP surgery; Q8: Understanding of the purpose of each step in PVP surgery; Q9: Capability of performing PVP surgery on a physical model; Q10: Expectation for further hands-on simulation training.

During this study, all students were taught theoretical concepts using slides and models of the human body. The augmented reality group then had percutaneous vertebroplasty surgical operation simulations and instruction using augmented reality models after receiving traditional instruction (Figures 5, 6). Teaching quality, students’ learning interest, anatomical structure mastery, and knowledge of percutaneous vertebroplasty were all significantly different between group A and group B, according to the results of the post-class evaluations. There were notable benefits (*p* < 0.05) for the group that was taught using augmented reality technology (Table 1). In addition, after finishing the regular academic offerings and the first teaching scenario survey, every participant in the traditional teaching group was also allowed to learn by AR simulation technology. A second survey with questions was administered following the completion of all training in the traditional teaching group (Group C). After implementing AR into the traditional teaching group, students demonstrated greater engagement in learning, enhanced comprehension of anatomical structures, and higher ratings of the overall quality of instruction (Table 2). Along with their proficiency in percutaneous vertebroplasty, students demonstrated considerable enhancements in their comprehension of the patterns of growth and spread of spinal malignancies (Table 2). The box plots were used to display the distribution among three groups (Figure 7).

**Table 1:** Comparison of course evaluations using two different teaching methods.

Teaching feedback items	Group A	Group B	U	p	r
	*N* = 13	*N* = 12			
1 Teaching quality	8.00 (7.75, 8.25)	9.00 (9.00, 10.00)	25.50	0.0033^**^	0.5872
2 Learning interest	7.00 (7.00, 8.00)	9.00 (8.00, 10.00)	28.00	0.0055^**^	0.5552
3 Understanding of spinal anatomy	7.00 (6.75, 7.25)	8.50 (8.00, 10.00)	17.50	0.0008^**^	0.6706
4 Understanding of spinal trabecular structure	7.00 (6.00, 8.00)	8.00 (8.00, 9.00)	40.50	0.0376^*^	0.416
5 Understanding of the common sites for spinal tumors	8.00 (7.00, 8.00)	8.50 (8.00, 9.00)	38.00	0.0201^*^	0.4648
6 Understanding of the involvement and metastasis of spinal tumors	8.00 (6.00, 8.00)	8.50 (7.00, 9.00)	51.50	0.1388	0.296
7 Understanding of the general steps of PVP surgery	8.00 (7.00, 8.00)	9.00 (8.00, 9.50)	18.50	0.0009^**^	0.6662
8 Understanding of the purpose of each step in PVP surgery	7.00 (6.00, 8.00)	9.00 (8.00, 10.00)	22.00	0.0021^**^	0.6164
9 Capability of performing PVP surgery on a physical model	4.00 (3.75, 6.25)	8.00 (4.50, 8.00)	42.50	0.0513	0.3898
10 Expectation for further hands-on simulation training	8.00 (6.00, 8.25)	9.00 (8.00, 10.00)	37.50	0.0256^*^	0.4464

Group A is the traditional teaching group at the first questionnaire survey; Group B is the augmented reality assisted teaching group; The data are described as the median and 25 and 75th interquartile ranges (Q25, Q75); The effect size (ES) was calculated by dividing the *Z*-score by the square root of *n*: 0.20–0.49 is considered a small effect; 0.50–0.79 is considered a medium effect; and 0.80 and above are considered a large effect.

**Table 2:** Comparison of course evaluations of students in the traditional teaching group before and after receiving augmented reality technology teaching.

Teaching feedback items	Group A	Group C	*Z*	*p*
	*N* = 13	*N* = 13		
1 Teaching quality	8.00 (7.75,8.25)	9.00 (8.75,10.00)	−3.071	0.002^**^
2 Learning interest	7.00 (7.00, 8.00)	9.00 (9.00,10.00)	−3.228	0.001^**^
3 Understanding of basic spinal anatomy	7.00 (6.75, 7.25)	9.00 (8.00,9.00)	−3.286	0.001^**^
4 Understanding of spinal trabecular structure	7.00 (6.00,8.00)	8.00 (8.00,9.50)	−2.734	0.006^**^
5 Understanding of the common sites for spinal tumors	8.00 (7.00, 8.00)	8.00 (8.00,9.00)	−3.000	0.003^**^
6 Understanding of the involvement and metastasis of spinal tumors	8.00 (6.00, 8.00)	9.00 (8.00,9.00)	−2.640	0.008^**^
7 Understanding of the general steps of PVP surgery	8.00 (7.00, 8.00)	9.00 (9.00,10.00)	−3.095	0.002^**^
8 Understanding of the purpose of each step in PVP surgery	7.00 (6.00, 8.00)	9.00 (8.00,10.00)	−3.169	0.002^**^
9 Understanding of performing PVP surgery on a physical model	4.00 (3.75,6.25)	6.00 (5.00, 8.00)	−2.994	0.003^**^
10 Expectations for further operational simulation training	8.00 (6.00,8.25)	9.00 (8.00,9.50)	−2.658	0.008^**^

Group A is the traditional teaching group at the first questionnaire survey; Group C is the traditional teaching group where students are re-evaluated for teaching feedback after receiving augmented reality teaching at the second questionnaire survey; The data are described as the median and 25th and 75th interquartile ranges (Q25, Q75).

### Opinions and feasible improvement measures proposed by students

According to the study above, the researchers collected the identified issues, methods for enhancement, and the teaching pros and cons for each set of test students by utilizing subjective feedback during the teaching process (Table 3). The survey questionnaires for each group are listed as attachment forms (Supplementary materials 1, 2). The findings indicated that using AR holographic glasses might lead to a higher incidence of discomfort among students, such as dizziness, while initially using them, primarily due to the weight of the helmet and the three-dimensional image. However, after making the required adjustments, discomfort can be quickly relieved.

**Table 3:** Summary of subjective opinions raised by students after two different teaching courses.

Feedback items	Group	
	A	B
1	Theoretical knowledge is dull.	More AR models will yield better results.
2	Insufficient multimedia materials	Very good, much clearer than looking at the general picture.
3	If teaching aids were available, the content might be more intuitive.	Hope to see the development of interactive surgical demonstrations in the future.
4	More discussion sections are needed.	Hope to see the development of more actionable AR content in the future.
5	If possible, including surgical illustration videos could yield better results.	The clarity needs improvement. Two students experienced dizziness during the initial test but were able to adapt within 1–2 min.

Group A is the traditional teaching group; Group B is the augmented reality (AR) assisted teaching group.

## Discussion

### Application of AR in medical education

Virtual reality (VR) technology has been instrumental in enhancing psychological skill training in high-performance professions, especially those that require a high level of safety awareness and complex skills (17). VR can efficiently train and enhance psychological skills by creating a safe, repeatable, and fully controllable training environment. In high-pressure and high-performance environments, the VR technology offers various innovative solutions for the training and learning of complex skills, which are often expensive or dangerous to practice in reality (18, 19). Extended reality (XR) is an advanced version and expansion of VR technology, and the utilization of XR in orthopedics and medical education is gaining popularity. AR technology emerges from integrating human visual exploration and interaction with computer-generated 3D worlds, which are then overlaid onto the real-world environment, incorporating virtual circumstances (20). In medical education, AR tools allow for anatomical simulation, evaluation, diagnosis, and surgical teaching and training through realistic surgical simulations (21). In recent years, the teaching attempts and applications of AR technology in undergraduate, graduate, and junior resident physicians have received immense attention (22).

Through the VR/AR technology, realistic operating environments can be simulated, enabling trainees to improve key skills in virtual contexts, such as attention, decision-making ability, situational awareness, adaptability, and stress management (18). Such signals can be monitored and evaluated by analyzing physiological parameters such as heart rate, respiratory rate, and electroencephalogram (EEG) (19, 23). By wearing AR glasses, students and teachers can learn about the anatomy, imaging knowledge, and surgical approach selection of relevant cases in space of virtual and real fusion. Applying this teaching method allows for a more intuitive and clear display of the anatomical structure of limbs, joints, and spine. Küçük et al. (24) found that the use of AR technology to learn anatomical knowledge has an impact on the academic performance and cognitive aspects of medical students. Lopes et al. (25) divided 20 medical students without any experience in basic surgical skills into an AR telestration teaching group and a traditional teaching group, and then evaluated the students’ learning experience, self-confidence, and self-assessment through questionnaires. Through result analysis, it was found that using helmet devices with AR functions can replace traditional teaching methods in terms of the quality of the surgical gesture, tension of the suture, self-evaluation or confidence. In addition, Nagayo et al. (26) established a self-training system that provides 3D information through AR technology. There was no significant difference in the improvement of test scores between the AR group and the video group, however, the actions provided by the AR system were more helpful in manipulating surgical instruments than videos (*p* = 0.02). Students can quickly grasp and apply knowledge points such as complex fracture, joint disorders, and spinal diseases. Meanwhile, it also frees medical education from the limitations of traditional teaching venues (22).

### Application of AR in teaching spinal tumor

Surgery for tumor in bone and soft tissue is a specialized area that combines the expertise of orthopedics and oncology. Subspecialty teaching encounters numerous hurdles, including intricate anatomical underpinnings, a wide range of disease classifications, and lengthy training periods for young physicians. Although the application of VR/AR/MR technology in orthopedic specialties such as spine, joints, and trauma has been on the rise (27, 28), few studies have explored the application of VR/AR/MR technology in bone and soft tissue tumors (Table 4). The management of bone tumors requires long-term and continuous clinical learning and practical operation. Therefore, the VR/AR/MR technology may provide simulated clinical teaching incorporating hearing, vision, and touch elements, enabling students to participate in clinical practice under various scenarios. It also facilitates real-time guidance from teachers, increases teacher-student interaction, and improves teaching efficiency.

**Table 4:** Summary of previous literature on the application of AR/MR/VR/XR technology in bone and soft tissue tumors.

Number	First author	Publication year	Application technology	Application field	Application effect	Specific anatomical site of application	Number of students	Application advantages	Application shortcomings
1	Caliskan et al. (29)	2024	Mixed reality cutting neuronavigation technology based on 3D printed markers.	Spine surgery field	It is clinically feasible and easy for surgeons to use, as well as being time-saving, cost-effective and highly precise.	C5-C6	Not mentioned in the article.	It is clinically feasible and easy for surgeons to use, as well as being time-saving, cost-effective and highly precise.	This technology has shortcomings in terms of not being suitable for emergency surgeries, system delays, limitations of equipment and materials, difficulty in popularizing the technology, accuracy, and intraoperative positioning.
						C7-T1			
						T5-T6			
2	Bian et al. (30)	2024	Review of extended reality (XR) technology, including virtual reality (VR), augmented reality (AR), and mixed reality (MR).	Intraoperative navigation in orthopedic surgery.	Improves accuracy of implant positioning, reduces post-operative complications, shortens operative time, and reduces radiation exposure to patients and surgeons.	Mainly used in trauma, joint, spine and bone tumor surgery.	Not mentioned in the article.	Improve surgical accuracy.	System accuracy needs to be further improved.
								Reduce surgical complications.	Equipment costs are high and still in the development stage.
								Shorten surgery time.	
								Reduce radiation exposure.	Lack of standardized norms and objective evaluation criteria.
3	Al-Naser et al. (31)	2024	Augmented reality (AR)	Image-guided tumor ablation	Improves the accuracy of ablation probe positioning and surgical efficiency, enables real-time guidance and enhanced visualization, and improves navigation capabilities.	Sites commonly involved in tumor ablation include bone, abdominal soft tissue, breast, liver, kidney, colorectum, and lungs.	Not mentioned in the article.	Reduced radiation exposure.	Small sample size, big technical challenges.
								Shortened operation time.	
								Improved navigation and target positioning assistance capabilities.	More research is needed to validate these findings.
4	Fan et al. (32)	2023	Medical image-guided orthopedic surgery (IGOS), including artificial intelligence (AI), deep learning (DL), augmented reality (AR), and robotics.	Orthopedic surgery	Through the application of these technologies, surgical risks are reduced and surgical results are improved.	Orthopedic related parts such as spine, joints, fracture sites and bone tumor areas.	Not mentioned in the article.	Improve surgical accuracy and efficiency, reduce intraoperative radiation exposure, and improve doctors’ navigation capabilities during surgery.	High heterogeneity in technology implementation, resulting in inconsistent application across institutions; lack of standardization of quantitative and technical requirements; further research and technological advancements are needed to overcome positioning errors.
5	D’Arienzo et al. (33)	2023	3D printing and augmented reality	Using 3D printing and augmented reality for preoperative planning, specifically in the cryotherapy of giant cell tumors, a mixed reality planning platform was created using 3D printing and augmented reality for precise placement of cryoprobes.	This technology increases the reliability of preoperative planning, is easily replicable, and provides surgeons with a practical tool.	Giant cell tumor of bone, especially the distal long bones, femur.	Not mentioned in the article.	Improves the accuracy of preoperative planning and ease of operation, and improves the surgeon’s awareness of the lesion and surrounding anatomy through augmented reality technology.	The number of patients in the current study is limited, and more clinical data are needed to verify these preliminary results.
6	Bruschi et al. (34)	2023	Patient Specific Instrumentation (PSI) and Intraoperative Surgical Navigation (SN)	Bone tumor resection, a review comparing the use of two techniques in tumor resection.	Both techniques have similar effectiveness in achieving wide tumor resection margins while sparing bone.	Long bones and pelvis	Not mentioned in the article.	Both techniques have similar effectiveness in achieving wide tumor resection margins, saving surgical time.	When resecting long bones, the accuracy of both techniques may be affected by surgical space limitations, with PSI requiring longer production time and SN requiring shorter operating time.
7	Wong et al. (35)	2023	Mixed Reality (MR)	Orthopedic oncology for surgical planning of bone tumors.	The use of MR technology improved 3D visualization and spatial awareness of bone tumors, facilitated surgical planning before skin incisions, and reduced the cognitive workload of surgeons during preoperative clinical assessment. Surgeons reported enhanced spatial understanding and found MR technology more effective compared to conventional 2D imaging methods.	Various locations including pelvis, tibia, proximal femur, scapula, proximal humerus, and calcaneus.	Not mentioned in the article.	Improved spatial awareness of tumors’ locations and orientations.	Small sample size (nine patients), limiting statistical significance.
								Reduced cognitive workload for surgeons.	Single surgeon experience, which may introduce bias.
								Enhanced preoperative planning and decision-making.	MR technology is still developing, and access to technology may be limited.
								Real-time interaction with 3D holograms.	Subjective assessment methods (Likert-Scale and NASA-TLX) may not fully capture the effectiveness of the technology.
								No discomfort reported by surgeons using MR technology.	Improvement in spatial awareness does not necessarily translate into better clinical outcomes, which need further investigation.
8	Tigchelaar et al. (36)	2022	Augmented reality (AR)	En bloc resection of spinal lesions with augmented reality-assisted neuronavigation, using the SyncAR system combined with a surgical microscope and navigation system to display 3D navigation data in real time directly in the surgeon’s field of view during the surgery.	Improved precision of surgical incisions, reduced bone resection, and enhanced visualization of key adjacent anatomy, and the patient recovered well post-operatively with no known complications.	Spine, mainly including the sacrum and thoracic vertebrae.	Not mentioned in the article.	Improves surgical precision, reduces surgical risks, and improves postoperative outcomes.	Augmented reality technology often faces challenges such as high cost, operational complexity, and technology acceptance.
9	Suzuki et al. (37)	2022	Augmented reality (AR)	Using augmented reality technology for non-contact measurement of puncture needle angles guided by computed tomography (CT), a non-contact angle measurement application was developed that utilized augmented reality platform software for real-time measurement of needle angles and optimized related Three-dimensional coordinate design.	In 18 tumor biopsy cases, the average difference between AR and CT measurement results was −0.2°, and the measurement accuracy was 2.0°.	Needle biopsy of renal, pulmonary, retroperitoneal, soft tissue, thyroid, mesenteric, and bone tumors.	Not mentioned in the article.	Provides a non-contact and real-time solution for accurately measuring puncture needle angle under CT guidance, improving puncture accuracy and efficiency.	Although augmented reality technology provides high-precision measurements, depending on software performance and algorithm optimization, the application effects may vary on different devices.
10	Ivanov et al. (38)	2022	Augmented reality and mixed reality technology (AR/MR)	Surgery for abdominal cancer patients	Improves surgical accuracy and safety, reduces surgical time and complications, and improves recovery period.	Abdomen	Not mentioned in the article.	Improve surgical accuracy, simplify surgical procedures, improve communication between doctors and patients, and improve surgical safety.	The technology relies heavily on equipment and needs further optimization to enhance positioning accuracy and reduce costs.
11	Rios-Vicil et al. (39)	2022	Extended Reality (XR), including Virtual Reality (VR) and Augmented Reality (AR)	Neurosurgery, specifically cranioplasty after tumor resection.	The XR-based workflow allowed for precise and efficient customization and placement of the implant, resulting in excellent cosmetic outcomes, reduced operative time, and a feasible surgical workflow. The technique avoided the common pitfalls of current single-stage workflows, such as increased operative times for tailoring implants and large incisions for accommodating templates.	Left frontal bone (skull)	Not mentioned in the article.	Improved precision in implant customization and placement.	High initial cost of implementing VR/AR platforms.
								Reduced operative time.	Learning curve associated with adopting new technology.
								Enhanced cosmetic outcomes.	Limited studies evaluating objective patient outcomes and patient satisfaction.
								Feasible and efficient surgical workflow.	Need for high-resolution imaging and dedicated personnel and space for VR/AR systems.
								Avoids large incisions and the need for extensive intraoperative implant modification.	
12	Zhang et al. (40)	2022	3D Reconstruction and Mixed Reality (MR)	Surgical oncology for resection of sacrococcygeal teratomas	The combination of 3D reconstruction and MR technology facilitated precise surgical planning and execution. It allowed the surgical team to visualize the relationship between the tumor and surrounding tissues, aiding in the complete resection of the teratoma without complications. The technology also helped in avoiding damage to vital structures like the gluteal artery, sciatic nerve, and rectal ampulla.	Sacrococcygeal region	Not mentioned in the article.	Enhanced preoperative planning with detailed spatial visualization of the tumor.	The accuracy of the MR system is limited by the resolution of CT imaging, which may not capture small tissues.
								Improved surgical precision and effectiveness.	Potential discrepancies between virtual and actual anatomical structures due to factors like hemorrhage.
								Reduced risk of complications and ensured complete tumor removal.	The need for postoperative monitoring to detect possible recurrence or malignant transformation.
								Facilitated patient understanding of the surgical procedure and associated risks.	Requires high-resolution imaging and specialized equipment, which may not be readily available in all clinical settings.
13	Moreta-Martinez et al. (41)	2021	Augmented reality (AR) and three-dimensional printing (3D Printing)	Orthopedic oncology surgery	Improved the accuracy of surgical navigation, optimized surgical procedures, and enhanced patient communication.	Involves a variety of orthopedic tumors, including tumors in different skeletal and anatomical regions.	Not mentioned in the article.	Improve surgical accuracy, improve patient experience, and simplify surgical planning.	For large anatomical models, 3D printing may require longer time and more materials, and there is still room for improvement in real-time data registration of augmented reality technology during surgery.
14	Faiella et al. (42)	2021	Augmented reality (AR)	Bone aspiration biologic examination and ablation treatment	Procedures using SIRIO navigation reduce the number of CT scans, shorten procedure times, and lower radiation dose to the patient than those with traditional CT navigation, especially when dealing with lesions less than 2 cm in diameter	Skeleton	Not mentioned in the article	Particularly effective when operating on small lesions, improving diagnostic accuracy and reducing radiation exposure	Requires specific equipment and software support, higher technical requirements for operators, and implementation costs may be higher
15	Abdel Al et al. (43)	2020	Augmented reality (AR)	Soft tissue sarcoma of foot	AR technology was successfully used to perform precise resection of foot tumors. The patient recovered well after surgery and his function and mobility were optimized.	Foot	Not mentioned in the article.	Improves surgical accuracy, improves surgical planning, enhances intraoperative visual navigation, and reduces surgical risks.	Reliance on technical assistants is high, and equipment needs to be continuously adjusted during surgery to ensure alignment of virtual and real images. Dependence on equipment and software may limit its widespread application.
16	Kaiser et al. (44)	2020	Augmented reality (AR)	Soft tissue sarcoma	Through the analysis of the impact of different body position changes on tumor location, the importance of patient position in preoperative planning is revealed, which has guiding significance for improving surgical accuracy and safety.	Lower limbs, especially thighs and calves.	Not mentioned in the article.	The application of augmented reality technology in soft tissue sarcoma surgery improves awareness of tumor location and surrounding structures, aiding preoperative planning and reducing intraoperative risks.	Accurate simulation and image acquisition of different body positions require high-precision technical support. The research is based on cadaver models, and the actual application effect requires further clinical verification.
17	Carl et al. (45)	2019	Augmented Reality (AR)	Surgery of intradural spinal tumors	AR supported surgery by visualizing the tumor outline and other relevant surrounding structures with high precision, as evidenced by an AR registration error of 0.72 ± 0.24 mm. This visualization facilitated the surgical process by providing an intuitive display of the tumor’s extent and surrounding anatomy.	Intradural spinal tumors, including the cervical and thoracic spine regions.	Not mentioned in the article.	Provides an intuitive visualization of the tumor extent and surrounding structures.	The AR visualization in the direct surgical microscope view is not yet optimal and sometimes obscures the clear view.
								Ensures high precision with an AR registration error of 0.72 ± 0.24 mm.	Requires better contrast adaptation and resolution of the HUD.
								Allows for smooth integration into the surgical workflow.	AR visualization needs to become more immersive to avoid the perception of being solely projected on top of reality.
								Reduces radiation exposure with low-dose CT scanning.	Limited by a small number of cases in the study and difficulty in proving the direct impact on surgical outcomes
								Enhances hand-eye coordination and depth perception during surgery.	
18	Cho et al. (46)	2018	Augmented reality (AR)	Pelvic bone tumor surgery	Augmented reality-assisted resection group performed better at achieving planned resection margins with smaller errors.	Pelvis	Not mentioned in the article.	Improve surgical accuracy, simplify operating procedures, and reduce the complexity and space occupied by traditional navigation systems.	Despite providing promising preliminary data, the technology requires further validation in live animal models and is not currently ready for clinical trials in humans.
19	Cho et al. (47)	2017	Augmented reality (AR)	Bone tumor removal surgery	Augmented reality navigation system improves surgical resection accuracy, with AR-assisted resection errors smaller than traditional methods.	Long bone (pork femur)	Not mentioned in the article.	Improves the accuracy of tumor resection surgery, simplifies the surgical process, and reduces complex settings and time costs during surgery.	The system is designed for long bones and specific software or equipment may be required for tumor resection in the pelvis or complex structures.
20	JingSheng Shi (48)	2014	Virtual Reality (VR)	Orthopedic oncology, specifically for periarticular tumors	The VR system allowed for accurate reconstruction of anatomical structures and tumor measurements, which were consistent with intraoperative findings. The virtual simulations helped in surgical planning by exposing lesion sites and generating individualized surgical plans. The system improved treatment individualization and surgical competence and potentially reduced intraoperative complications.	Periarticular regions including the proximal femur, proximal tibia, proximal humerus, and distal tibia.	Not mentioned in the article.	Accurate reconstruction of anatomical structures.	Small sample size (10 cases), limiting statistical analysis and generalizability.
								Improved surgical planning and treatment individualization.	Discrepancies between virtual and actual surgical situations in some cases.
								Enhanced surgical competence and reduced intraoperative complications.	Limited visualization quality for soft tissues.
								Consistent measurements of tumor size between virtual and actual data.	The system is not specifically designed for orthopedic surgery and lacks corresponding haptic feedback.
									Further specialization of VR tools and validation methodologies for orthopedic applications are needed.

AR, Augmented reality; MR, Mixed reality; VR, Virtual reality; XR, Extended reality.

Spinal tumor surgery poses significant challenges and risks, particularly in cases of malignant tumors, when determining the tumor boundaries is typically challenging. The surgical procedure is challenging and demands exceptional surgical expertise from the operator. In this context, spinal tumors, as one of the most difficult bone tumor diseases in orthopedics, have the characteristics of difficult-to-grasp anatomical structures, difficult-to-understand tumor invasion characteristics, and long surgical learning curves. As an innovative teaching method, AR technology has rapidly developed with unique advantages such as immersion, interactivity, and accuracy, helping to promote the transformation and progress of teaching models in bone and soft tissue tumor surgery. To assure surgical safety, AR technology can visualize crucial anatomical structures, including main blood arteries, nerves, spinal cord, and other vital tissues. The time needed for surgical procedures can be significantly decreased by projecting these rebuilt anatomical structures onto the patient (47). Under such circumstances, this study explores the training effectiveness of applying augmented reality technology in simulated teaching of spinal tumors.

Cho et al. was the first to report the application of AR-assisted navigation technology in surgical resection of pig femoral and pelvic tumors, laying the foundation for the application of AR technology in the field of bone and soft tissue tumors (46, 47). Subsequently, Carl et al. (45) and Tigchelaar et al. (36) applied AR technology to the surgical treatment of spinal intradural tumor and spinal epidural tumor, respectively. However, although there are many literature reports, the expansion and attempts of AR-assisted teaching methods for medical education are especially lacking. In this context, our team developed an AR-assisted spinal tumor-teaching model and surgical teaching system for medical students.

### The main contribution of this study

This study was the first reported attempt to apply AR technology in medical teaching, using spinal tumors and percutaneous vertebroplasty as examples, head-mounted augmented reality devices were used by students to create specific learning scenarios, presenting the complex three-dimensional spatial structure intuitively. After further data analysis, we found that the AR-assisted teaching group had better teaching quality in learning interest, understanding of spinal analysis, understanding of spinal trabecular structure, understanding of the common sites for spinal tubers, and understanding of the general steps of PVP surgery, compared to the traditional teaching group. In addition, after receiving AR-assisted teaching in traditional teaching group, significant improvements had been found in teaching quality, learning interest, understanding of basic spinal anatomy, understanding of spinal trabecular structure, understanding of the common sites for spinal tumors, understanding of the involvement and metathesis of spinal tumors, understanding of the general steps of PVP surgery, and expectations for further operational simulation training. The above research results confirmed the teaching significance of the spinal tumor model constructed in this study and the demonstration model of percutaneous vertebroplasty surgery, as well as the important value of augmented reality technology in assisting spinal tumor teaching. The research results of this study will lay a theoretical and practical foundation for the application of AR-assisted spinal tumor anatomy teaching and preclinical teaching, as well as the improvement of medical education level.

### Limitations and future plans

Spinal tumors are a difficult point for medical students or young physicians during learning, most of them require prolonged learning period to master. The use of AR technology to simulate spinal tumor models and interactive surgical scenarios needs to be further verified in the medical preparatory stage or young physicians. For this purpose, our research team independently designed a spinal tumor model and a percutaneous vertebroplasty surgery procedure that can be used to achieve interactive teaching. Although the number of medical students included in the two groups was not high, statistically significant differences were observed between multiple indicators among groups. Future research will focus on expanding the participant pool, exploring diverse teaching methods and scenarios, and incorporating objective skill assessments (e.g., surgical operation simulations) with scoring systems. As the number of students increases, more teaching data will be accumulated for more in-depth analysis of multiple factors such as gender, age, student type, and major. In terms of device improvement, students will have more opportunities for interactive operations to enhance their clinical operational skills. These advancements will further validate the feasibility of AR as a valuable tool for modern orthopedic and surgical oncology education.

Machine learning and artificial intelligence technology can be applied in psychological skill assessment and training, and the combination of the two is expected to further improve the quality of medical education (18, 23). For example, using machine learning algorithms, patterns can be extracted from a large amount of physiological and behavioral data to evaluate individual performance and progress at different training stages (23). The technologies can also be used to establish adaptive learning systems that dynamically adjust training content and difficulty based on real-time feedback from trainees, thereby improving training effectiveness. In addition, artificial intelligence can create virtual coaches and intelligent decision support systems, which may improve the experience of trainees in virtual reality learning environments (49, 50). Thus, the promotion and improvement of AR technology in medical teaching need to accumulate more practical data and still face many challenges (51, 52).

## Conclusion

In summary, AR technology, which combines reality and virtual worlds, has great potential in applying medical education. For teaching spinal tumors, it can effectively compensate for the shortcomings of traditional teaching models, make medical education more precise, efficient, and intuitive, and gradually promote the reform of medical education models. In clinical teaching of spinal tumors, AR-assisted teaching is an optimization method that has better teaching effectiveness and can be further explored in the future.

## Data availability statement

The original contributions presented in the study are included in the article/Supplementary material; further inquiries can be directed to the corresponding authors.

## Ethics statement

The ethical approval of this study was evaluated and waived by the Ethics Committee of Peking Union Medical College Hospital (No. I-24ZM0019). The authors confirm that all methods were carried out in accordance with relevant guidelines and regulations. Written informed consent was obtained from all subjects involved in this study.

## Author contributions

SLiu: Conceptualization, Formal analysis, Funding acquisition, Investigation, Methodology, Resources, Software, Writing – original draft, Writing – review & editing. JY: Conceptualization, Formal analysis, Investigation, Methodology, Project administration, Resources, Software, Writing – original draft, Writing – review & editing. HJ: Conceptualization, Formal analysis, Methodology, Resources, Software, Writing – original draft. AL: Data curation, Investigation, Software, Writing – original draft. QZ: Conceptualization, Methodology, Resources, Software, Writing – original draft. JX: Data curation, Investigation, Software, Writing – original draft. YL: Conceptualization, Formal analysis, Funding acquisition, Investigation, Methodology, Project administration, Resources, Software, Supervision, Writing – original draft, Writing – review & editing. SLi: Conceptualization, Formal analysis, Investigation, Methodology, Project administration, Resources, Software, Supervision, Writing – original draft, Writing – review & editing.
