# Supplementary material for: Exploration of the application of augmented reality technology for teaching spinal tumor’s anatomy and surgical techniques
Source: Front Med (Lausanne). 2024 Jul 10;11:1403423. doi: 10.3389/fmed.2024.1403423 (PMC11266009; doi:10.3389/fmed.2024.1403423)
Supplement: Supplementary file 1 [file Table_1.docx]

**Course Feedback Survey A (English version)**

The data collected from this post-course survey will be used for data analysis and statistical purposes to explore more scientific and effective educational teaching methods. Thank you for your active cooperation, and we wish you a successful academic journey!

Basic Information

- Age: (e.g., 24 years old)

___________________________________

- Gender: (Male/Female)

___________________________________

- Year of Entry: (e.g., 2022)

___________________________________

- Class:

___________________________________

- Are you an intern? [Single-choice question]

○ Yes

○ No

- Are you on a practicum? [Single-choice question]

○ Yes

○ No

**The following questions are represented on a 5-point Likert scale:**

- Very Satisfied (Score = 5)

- Satisfied (Score = 4)

- Neutral (Score = 3)

- Dissatisfied (Score = 2)

- Very Dissatisfied (Score = 1)

1. Does the instructor exhibit willingness and enthusiasm to teach, and do they proactively engage in teaching? [ ]

2. Does this course provide targeted instruction? [ ]

3. Does this course offer effective and timely feedback to students' questions? [ ]

4. Does the instructor have a solid and extensive theoretical foundation, and do they deliver instruction aligned with the students' knowledge level? [ ]

5. Does this course emphasize fostering students' initiative and interest in learning, encouraging questions and discussions? [ ]

6. Are you overall very satisfied with this course? [ ]

7. Is this course overall very valuable to you? [ ]

8. Has this course made it easier and clearer for you to develop a three-dimensional understanding of spinal anatomy and become familiar with surgical procedures? [ ]

9. Were you able to concentrate and naturally immerse yourself in the learning process during the course? [ ]

10. Does this course employ a variety of teaching methods that guide students towards exploratory, interactive, and participatory learning? [ ]

11. Does this course utilize teaching strategies, such as creating scenarios, to stimulate your interest in learning and promote active participation? [ ]

12. Does this course guide you to fully utilize course resources for self-directed learning, thereby enhancing your awareness and ability for self-directed learning? [ ]

13. Has this course enabled you to easily and intuitively grasp knowledge about spinal anatomy and the diagnosis and treatment of spinal tumors? [ ]

14. Through this course, have you gained a clear understanding and recognition of the three-dimensional structure of the spine, the process of percutaneous vertebroplasty, and surgical approaches? [ ]

15. Has this course improved your ability to analyze and solve problems? [ ]

16. Is the teaching content of this course primarily based on lecture notes or courseware, and is the course content monotonous and boring? [ ]

17. Is the teaching mode of this course innovative, with rich teaching resources to meet your diverse and personalized learning needs? [ ]

18. Are you very willing to recommend this course to your peers? [ ]

**Open-ended Questions**

**Please provide detailed answers to the following questions:**

1. What aspects of this course were most helpful to you?

___________________________________

2. What aspects of this course need improvement? What are your suggestions?

___________________________________

3. Do you have any other questions regarding this course?

___________________________________

4. Have you ever been exposed to Virtual Reality (VR), Augmented Reality (AR), or Mixed Reality (MR)? How was your experience?

- Have you ever been exposed? [Single-choice question]

○ Yes

○ No

5. What are your thoughts on the application of MR technology in medical education?

___________________________________

**Note:** The data collected from this post-course survey will be used for data analysis and statistical purposes to explore more scientific and effective educational teaching methods. Thank you for your active cooperation, and we wish you a successful academic journey!

After the completion of the regular academic offerings and the completion of the first survey questionnaire, all students in group A received augmented reality teaching (the same content as group B). After completing all learning content, students in group A received the second survey.

**Please rate the following on a scale of 1 to 10:**

- (1) 1 point

- (2) 2 points

- (3) 3 points

- (4) 4 points

- (5) 5 points

- (6) 6 points

- (7) 7 points

- (8) 8 points

- (9) 9 points

- (10) 10 points

1. Please rate the quality of teaching using the traditional slide presentation method.

2. Please rate the quality of teaching using the traditional slide presentation method combined with actual anatomical model demonstrations.

3. Please rate the quality of teaching using the traditional slide presentation method combined with actual anatomical model demonstrations and mixed reality technology.

4. Please rate your interest in learning when the traditional slide presentation method is used.

5. Please rate your interest in learning when the traditional slide presentation method combined with actual anatomical model demonstrations is used.

6. Please rate your interest in learning when the traditional slide presentation method combined with actual anatomical model demonstrations and mixed reality technology is used.

7. Rate your understanding of the basic anatomical structure of the spine when the traditional slide presentation method is used.

8. Rate your understanding of the basic anatomical structure of the spine when the traditional slide presentation method combined with actual anatomical model demonstrations is used.

9. Rate your understanding of the basic anatomical structure of the spine when the traditional slide presentation method combined with actual anatomical model demonstrations and mixed reality technology is used.

10. Rate your understanding of the spinal cancellous bone and trabecular structure when the traditional slide presentation method is used.

11. Rate your understanding of the spinal cancellous bone and trabecular structure when the traditional slide presentation method combined with actual anatomical model demonstrations is used.

12. Rate your understanding of the spinal cancellous bone and trabecular structure when the traditional slide presentation method combined with actual anatomical model demonstrations and mixed reality technology is used.

13. Rate your understanding of the common sites of spinal tumor occurrence when the traditional slide presentation method is used.

14. Rate your understanding of the common sites of spinal tumor occurrence when the traditional slide presentation method combined with actual anatomical model demonstrations is used.

15. Rate your understanding of the common sites of spinal tumor occurrence when the traditional slide presentation method combined with actual anatomical model demonstrations and mixed reality technology is used.

16. Rate your understanding of the involvement and metastasis methods of spinal tumors when the traditional slide presentation method is used.

17. Rate your understanding of the involvement and metastasis methods of spinal tumors when the traditional slide presentation method combined with actual anatomical model demonstrations is used.

18. Rate your understanding of the involvement and metastasis methods of spinal tumors when the traditional slide presentation method combined with actual anatomical model demonstrations and mixed reality technology is used.

19. Rate your understanding of the general steps of percutaneous vertebroplasty (PVP) surgery when the traditional slide presentation method is used.

20. Rate your understanding of the general steps of percutaneous vertebroplasty (PVP) surgery when the traditional slide presentation method combined with actual anatomical model demonstrations is used.

21. Rate your understanding of the general steps of percutaneous vertebroplasty (PVP) surgery when the traditional slide presentation method combined with actual anatomical model demonstrations and mixed reality technology is used.

22. Rate your understanding of the purpose of each step of percutaneous vertebroplasty (PVP) surgery when the traditional slide presentation method is used.

23. Rate your understanding of the purpose of each step of percutaneous vertebroplasty (PVP) surgery when the traditional slide presentation method combined with actual anatomical model demonstrations is used.

24. Rate your understanding of the purpose of each step of percutaneous vertebroplasty (PVP) surgery when the traditional slide presentation method combined with actual anatomical model demonstrations and mixed reality technology is used.

25. Do you feel capable of performing percutaneous vertebroplasty (PVP) surgery on a physical model after the class ends when the traditional slide presentation method is used?

26. Do you feel capable of performing percutaneous vertebroplasty (PVP) surgery on a physical model after the class ends when the traditional slide presentation method combined with actual anatomical model demonstrations is used?

27. Do you feel capable of performing percutaneous vertebroplasty (PVP) surgery on a physical model after the class ends when the traditional slide presentation method combined with actual anatomical model demonstrations and mixed reality technology is used?

28. What is your expectation for further hands-on model simulation training when only the traditional slide presentation method is used?

29. What is your expectation for further hands-on model simulation training when the traditional slide presentation method combined with actual anatomical model demonstrations is used?

30. What is your expectation for further hands-on model simulation training when the traditional slide presentation method combined with actual anatomical model demonstrations and mixed reality technology is used?

**Note:** The data collected from this post-course survey will be used for data analysis and statistical purposes to explore more scientific and effective educational teaching methods. Thank you for your active cooperation, and we wish you a successful academic journey!
