# Supplementary material for: Exploration of the application of augmented reality technology for teaching spinal tumor’s anatomy and surgical techniques
Source: Front Med (Lausanne). 2024 Jul 10;11:1403423. doi: 10.3389/fmed.2024.1403423 (PMC11266009; doi:10.3389/fmed.2024.1403423)
Supplement: Supplementary file 2 [file Table_2.docx]

**Course Feedback Survey B (English version)**

The data collected from this post-course survey will be used for data analysis and statistical purposes to explore more scientific and effective educational teaching methods. Thank you for your active cooperation, and we wish you a successful academic journey!

Basic Information

- Age: (e.g., 24 years old)

___________________________________

- Gender: (Male/Female)

___________________________________

- Year of Entry: (e.g., 2022)

___________________________________

- Class:

___________________________________

- Are you an intern? [Single-choice question]

○ Yes

○ No

- Are you on a practicum? [Single-choice question]

○ Yes

○ No

**The following questions are represented on a 5-point Likert scale:**

- Very Satisfied (Score = 5)

- Satisfied (Score = 4)

- Neutral (Score = 3)

- Dissatisfied (Score = 2)

- Very Dissatisfied (Score = 1)

1. Does the instructor exhibit willingness and enthusiasm to teach, and do they proactively engage in teaching? [ ]

2. Does this course provide targeted instruction? [ ]

3. Does this course offer effective and timely feedback to students' questions? [ ]

4. Does the instructor have a solid and extensive theoretical foundation, and do they deliver instruction aligned with the students' knowledge level? [ ]

5. Does this course emphasize fostering students' initiative and interest in learning, encouraging questions and discussions? [ ]

6. Are you overall very satisfied with this course? [ ]

7. Is this course overall very valuable to you? [ ]

8. Has this course made it easier and clearer for you to develop a three-dimensional understanding of spinal anatomy and become familiar with surgical procedures? [ ]

9. Were you able to concentrate and naturally immerse yourself in the learning process during the course? [ ]

10. Does this course employ a variety of teaching methods that guide students towards exploratory, interactive, and participatory learning? [ ]

11. Is this course adept at using teaching strategies such as scenario creation to stimulate your interest in learning and promote active participation? [ ]

12. Does this course guide you to fully utilize course resources for self-directed learning, thereby enhancing your awareness and ability for self-directed learning? [ ]

13. Has this course enabled you to easily and intuitively grasp knowledge about spinal anatomy and the diagnosis and treatment of spinal tumors? [ ]

14. Through this course, have you gained a clear understanding and recognition of the three-dimensional structure of the spine, the process of percutaneous vertebroplasty, and surgical approaches? [ ]

15. Has this course improved your ability to analyze and solve problems? [ ]

16. Is the teaching content of this course primarily based on lecture notes or courseware, and is the course content monotonous and boring? [ ]

17. Is the teaching mode of this course innovative, with rich teaching resources to meet your diverse and personalized learning needs? [ ]

18. Are you very willing to recommend this course to your peers? [ ]

19. How satisfied are you with the Mixed Reality (MR) teaching model? [ ]

20. Do you find the device operation easy to learn and master during the course? [ ]

21. Does the interface design conform to your usage habits during the learning process? [ ]

22. Are the three-dimensional models of bone and soft tissue tumors clear and structurally accurate? [ ]

23. Are the surgical demonstration processes clear and intuitive, aiding your understanding of surgical principles and basic surgical techniques? [ ]

24. Are you satisfied with the three-dimensional interactive experience provided by mixed reality technology? [ ]

25. Do you experience any discomfort such as dizziness, dry eyes, or neck and shoulder fatigue during the learning process? [ ]

26. If discomfort occurs, how long after use do the symptoms appear? (Please specify the duration in minutes) ___________________________________

27. Do you find the glasses heavy? [ ]

28. Are you satisfied with the image clarity during actual operation? [ ]

29. Do you believe mixed reality technology can be effectively used in undergraduate medical education? [ ]

30. Do you feel confident in mastering the required operations during the learning process? [ ]

31. Do you find the operation of the bone and soft tissue tumor model efficient? [ ]

32. Does the interface design conform to your usage habits during the learning process? [ ]

33. Are the anatomical structures presented easier to understand and recognize? [ ]

34. How accurate are the three-dimensional models of bone and soft tissue tumors compared to actual anatomical structures? [ ]

35. Do you think the accuracy of the simulated anatomical structures is sufficient to support actual surgical operations? [ ]

36. Are you satisfied with the three-dimensional interactive experience provided by mixed reality technology? [ ]

37. Do you think the three-dimensional interactive experience enhances your understanding and learning of bone and soft tissue tumors? [ ]

38. In the classroom, did you feel a more positive and pleasant learning atmosphere compared to traditional teaching? [ ]

39. In the classroom, were you more willing to ask questions, participate in discussions, and share opinions? [ ]

40. Do you believe mixed reality technology has encouraged you to participate more actively in the theoretical study of bone and soft tissue tumors? [ ]

41. Has your interest in the theoretical study of bone and soft tissue tumors increased through the use of mixed reality technology? [ ]

42. Does mixed reality technology help you focus more on the class content? [ ]

43. Is it easier to master the theoretical knowledge of bone and soft tissue tumors using mixed reality technology in the classroom? [ ]

44. Do you think mixed reality technology is effective in helping you understand and remember the anatomical structures and related knowledge of bone and soft tissue tumors? [ ]

45. How do the simulated three-dimensional anatomical structures compare to actual patients or specimens when using mixed reality technology? [ ]

46. Is it easier to apply theoretical knowledge to actual cases using mixed reality technology? [ ]

47. Do you think mixed reality technology is worth widely applying in future teaching, including theoretical instruction, clinical education, preoperative planning, and preoperative simulation training for bone and soft tissue tumors? [ ]

**Open-ended Questions**

**Please provide detailed answers to the following questions:**

1. What improvements do you think are needed for the MR teaching model, and what are your suggestions?

___________________________________

2. What aspects of the MR device display model and surgical video need enhancement?

___________________________________

3. Did you experience any discomfort such as dizziness, dry eyes, or neck and shoulder fatigue while using the MR device? What methods did you find helpful for relief?

___________________________________

4. Please share your personal learning experience using mixed reality technology for the theoretical study of bone and soft tissue tumors.

___________________________________

**Please rate the following on a scale of 1 to 10:**

- (1) 1 point

- (2) 2 points

- (3) 3 points

- (4) 4 points

- (5) 5 points

- (6) 6 points

- (7) 7 points

- (8) 8 points

- (9) 9 points

- (10) 10 points

1. Please rate the quality of teaching using the traditional slide presentation method.

2. Please rate the quality of teaching using the traditional slide presentation method combined with actual anatomical model demonstrations.

3. Please rate the quality of teaching using the traditional slide presentation method combined with actual anatomical model demonstrations and mixed reality technology.

4. Please rate your interest in learning when the traditional slide presentation method is used.

5. Please rate your interest in learning when the traditional slide presentation method combined with actual anatomical model demonstrations is used.

6. Please rate your interest in learning when the traditional slide presentation method combined with actual anatomical model demonstrations and mixed reality technology is used.

7. Rate your understanding of the basic anatomical structure of the spine when the traditional slide presentation method is used.

8. Rate your understanding of the basic anatomical structure of the spine when the traditional slide presentation method combined with actual anatomical model demonstrations is used.

9. Rate your understanding of the basic anatomical structure of the spine when the traditional slide presentation method combined with actual anatomical model demonstrations and mixed reality technology is used.

10. Rate your understanding of the spinal cancellous bone and trabecular structure when the traditional slide presentation method is used.

11. Rate your understanding of the spinal cancellous bone and trabecular structure when the traditional slide presentation method combined with actual anatomical model demonstrations is used.

12. Rate your understanding of the spinal cancellous bone and trabecular structure when the traditional slide presentation method combined with actual anatomical model demonstrations and mixed reality technology is used.

13. Rate your understanding of the common sites of spinal tumor occurrence when the traditional slide presentation method is used.

14. Rate your understanding of the common sites of spinal tumor occurrence when the traditional slide presentation method combined with actual anatomical model demonstrations is used.

15. Rate your understanding of the common sites of spinal tumor occurrence when the traditional slide presentation method combined with actual anatomical model demonstrations and mixed reality technology is used.

16. Rate your understanding of the involvement and metastasis methods of spinal tumors when the traditional slide presentation method is used.

17. Rate your understanding of the involvement and metastasis methods of spinal tumors when the traditional slide presentation method combined with actual anatomical model demonstrations is used.

18. Rate your understanding of the involvement and metastasis methods of spinal tumors when the traditional slide presentation method combined with actual anatomical model demonstrations and mixed reality technology is used.

19. Rate your understanding of the general steps of percutaneous vertebroplasty (PVP) surgery when the traditional slide presentation method is used.

20. Rate your understanding of the general steps of percutaneous vertebroplasty (PVP) surgery when the traditional slide presentation method combined with actual anatomical model demonstrations is used.

21. Rate your understanding of the general steps of percutaneous vertebroplasty (PVP) surgery when the traditional slide presentation method combined with actual anatomical model demonstrations and mixed reality technology is used.

22. Rate your understanding of the purpose of each step of percutaneous vertebroplasty (PVP) surgery when the traditional slide presentation method is used.

23. Rate your understanding of the purpose of each step of percutaneous vertebroplasty (PVP) surgery when the traditional slide presentation method combined with actual anatomical model demonstrations is used.

24. Rate your understanding of the purpose of each step of percutaneous vertebroplasty (PVP) surgery when the traditional slide presentation method combined with actual anatomical model demonstrations and mixed reality technology is used.

25. Do you feel capable of performing percutaneous vertebroplasty (PVP) surgery on a physical model after the class ends when the traditional slide presentation method is used?

26. Do you feel capable of performing percutaneous vertebroplasty (PVP) surgery on a physical model after the class ends when the traditional slide presentation method combined with actual anatomical model demonstrations is used?

27. Do you feel capable of performing percutaneous vertebroplasty (PVP) surgery on a physical model after the class ends when the traditional slide presentation method combined with actual anatomical model demonstrations and mixed reality technology is used?

28. What is your expectation for further hands-on model simulation training when only the traditional slide presentation method is used?

29. What is your expectation for further hands-on model simulation training when the traditional slide presentation method combined with actual anatomical model demonstrations is used?

30. What is your expectation for further hands-on model simulation training when the traditional slide presentation method combined with actual anatomical model demonstrations and mixed reality technology is used?

**Note:** The data collected from this post-course survey will be used for data analysis and statistical purposes to explore more scientific and effective educational teaching methods. Thank you for your active cooperation, and we wish you a successful academic journey!
